# To Be or Not to Be: The Case of Endoplasmic Reticulum Aminopeptidase 2

**DOI:** 10.3389/fimmu.2022.902567

**Published:** 2022-06-13

**Authors:** Piotr Kuśnierczyk

**Affiliations:** Laboratory of Immunogenetics and Tissue Immunology, Hirszfeld Institute of Immunology and Experimental Therapy, Polish Academy of Sciences, Wrocław, Poland

**Keywords:** ERAP2, genetics, peptide trimming, interactions with ERAP1, polymorphism, isoforms, endoplasmic reticulum aminopeptidase, ERAP1 interaction

## Abstract

To be, or not to be, that is the question. (William Shakespeare, Hamlet)

Endoplasmic reticulum aminopeptidases 1 and 2 (ERAP1 and ERAP2, respectively) play a role in trimming peptides that are too long to be bound and presented by class I HLA (HLA-I) molecules to CD8^+^ T cells. They may also affect the HLA-I-presented peptide repertoire by overtrimming potential epitopes. Both enzymes may also be released from the cell to cleave cytokine receptors and regulate blood pressure. Both enzymes are polymorphic, which affects their expression, specificity, and activity, resulting in their role in diseases associated with HLA-I. In this brief review, we concentrate on ERAP2, less investigated because of its lack in laboratory mice and 25% of humans, as well as a lower polymorphism. ERAP2 was found to be associated with several diseases and to influence ERAP1 effects. It was discovered recently that the defective *ERAP2* gene, not encoding functional aminopeptidase, may nevertheless, during viral infections, produce a truncated protein isoform of unknown function, possibly interfering with ERAP1 and full-length ERAP2 by heterodimer formation. The disease associations of ERAP2, alone or in combination with ERAP1, are reviewed.

## 1 Introduction

The human body is constantly exposed to infections with viruses, bacteria, fungi, and parasites. It is also confronted with arising neoplastically transformed self-cells. Therefore, it needs a defense that is provided by two branches of immunity: (i) innate, which is fast-acting but much less specific; and (ii) adoptive, which develops slower but is antigen-specific, i.e., focused on the given pathogen without being distracted by other, innocent structures. Both branches of immunity recognize foreign structures using proper receptors: In innate immunity, these are pattern-recognition receptors recognizing pathogen-associated molecular patterns, Toll-like receptors, distributed on several cell types, and killer cell immunoglobulin-like receptors (KIRs), the latter present on natural killer (NK) cells and subsets of T lymphocytes. KIRs recognize a lack or decrease in expression of human leukocyte antigen class I (HLA-I) molecules on the surface of target cells, and HLA-I-bound peptides influence this interaction (1–6). Adoptive immunity uses highly specific T-cell receptors (TCRs) that recognize antigenic peptides (derived from foreign or neoplastic proteins) presented to them by HLA molecules. Class I HLA molecules present intracellular antigens to TCRs of cytotoxic T cells (CD8+) which kill infected or transformed cells producing virus or tumor antigens, respectively. Both virus-infected cells and tumor cells frequently have decreased expression of cell surface HLA-I molecules to escape T-cell killing (7). Thus, both NK and T cells may eliminate a source of infection or malignant tumor, depending on HLA-I expression. HLA-I molecules bind their peptide cargo (8–10 amino acid long) in endoplasmic reticulum (ER). Peptides longer than 10 amino acids are trimmed by endoplasmic reticulum aminopeptidases to fit the peptide-binding groove of the HLA-I molecule. Class II HLA (HLA-II) molecules present extracellular antigens to helper T cells (CD4+). These then secrete cytokines and stimulate B lymphocytes to produce antigen-specific antibodies, recognizing antigens on viruses, bacteria, fungi, and parasites (8).

## 2 Endoplasmic Reticulum Aminopeptidases

Aminopeptidases are exopeptidases that hydrolyze amino acid residues from the N-terminus of peptides or protein substrates (9). M1 aminopeptidases (12 enzymes in humans) require Zn^2+^ for hydrolysis (10). Endoplasmic reticulum aminopeptidases 1 and 2 (ERAP1 and ERAP2, respectively) belong to the oxytocinase subfamily of M1 aminopeptidases together with insulin-responsive aminopeptidase (IRAP)^1^, the latter being present in intracellular vesicles including endosomes and engaged in cross-presentation, i.e., unconventional presentation of extracellular antigens by HLA-I molecules, and several other functions (11–13). These proteins are composed of four domains. In domain I, they contain an amino acid residue interacting with N-terminal residue of the peptidic substrate. Domain II contains two amino acid motifs important for the enzyme’s function: GAMEN is responsible for exopeptidase specificity by interaction with N-terminal amino acid of the trimmed peptide; Gln181 also contributes to the specificity. HELAH(X)_18_D is a zinc ligand and is essential for the catalytic activity of the enzymes. Domain III has a β-sandwich structure that links domains II and IV; it rotates in relation to domain II, forming an open and closed conformation of the molecule. Domain IV is a bowl that forms most of the large cavity accommodating the substrate. The enzyme is active in the closed conformation and may release the trimmed peptide and bind a new substrate in the open conformation (10, 14; and references therein).

ERAP1, in addition to the catalytic site, has a regulatory site distinct from but not far from the active site. Binding of the C-terminus of the peptide (in the open conformation) to a regulatory site promotes a change to a closed conformation with increased enzymatic activity. This mechanism provides trimming peptides of 9–16 amino acids in length but not shorter ones, which do not reach the regulatory site (“molecular ruler” model) (15 and references therein; 16). In spite of nearly 50% shared identity and a similar four-domain structure, ERAP2 is different from ERAP1 by a lack of this regulatory site binding the peptide C-terminus. This structural feature enables ERAP2 to digest octamers and shorter peptides but not so efficiently longer ones (15, 17; and references therein). Therefore, the effects of ERAP1 and ERAP2 on immunopeptidomes of particular HLA-I allotypes are different (18 and references therein). Nevertheless, using cells with different ERAP1 allotypes of high or low activity and expressing ERAP2 or not, it was observed that a remarkable part of immunopeptidome remains untouched by these changes (19, 20; and references therein).

Genes for three aminopeptidases are present, in different combinations, in vertebrates. Most mammals express ERAP1 and ERAP2 except for rodents, which are negative for ERAP2; virtually, all mammals have IRAP (21). In humans, ERAP1 and ERAP2 are highly (49% identity) homologous (11) but differ in their specificity: ERAP1 preferentially trims N-terminal hydrophobic amino acid residues, while ERAP2 prefers positively charged residues, particularly arginine (22).

In addition to their role in the endoplasmic reticulum, both enzymes may be released from the cell, where they may (i) perform cleavage of cell surface receptors for many cytokines, thus modulating immune responses (23) and (ii) regulate blood pressure (see *ERAPS in the Modulation of Cytokine-Mediated Signaling, in the Regulation of Blood Pressure, and in Sars-Cov-2 and HIV Infection*).

ERAP1 is highly polymorphic. Ten allotypes with frequencies at or above 0.4% were described in Europeans (24), and most of them are present also in other populations (25). The frequencies of these allotypes are different in distinct human populations and parallel the differences in HLA-I allele frequencies there. This may reflect the exposure of these populations to distinct pathogens in their environment. Interestingly, for example, homozygotes of ERAP1 allotype No. 10, of the lowest enzymatic activity, are absent or detected in only trace amounts in African, Asian, and Amerindian populations but are present in nearly 5% of Europeans (25). Allotypes of ERAP1 were reproducibly reported to be associated with those human diseases where HLA-I plays a role. These associations were reviewed recently (14, 18, 19, 23, 24, 26) and are out of the scope of the present article. It should be noted here that a remarkable fraction of peptides transported to the endoplasmic reticulum do not require any ERAP1-mediated pretreatment to be bound by HLA-I (20). At least some of them may be trimmed by ERAP2, and this may be a reason for the frequent coexistence of active ERAP2 with low activity ERAP1 in a haplotype (25, 27) (see the next section).

ERAP2 was less extensively examined because it is absent in 25% of humans and in mice, which are an important *in vivo* model for immunologic studies. However, it is also polymorphic, although to a lower extent (see the next section).

## 3 Polymorphism of *ERAP2* Gene—Apparent Lack of ERAP2 Protein Expression From Allotype B (rs2248374G)

*ERAP2* is less polymorphic than ERAP1, and most single nucleotide polymorphisms (SNPs) in this gene are in strong linkage disequilibrium (LD). This leads to two main allotypes differing in rs2248374A>G SNP, distributed at roughly equal frequencies in human populations (allotype A, rs2248374A, and allotype B, rs2248374G), and several minor variants of these two. Individuals homozygotic or heterozygotic for rs2248374A express functional ERAP2 protein, whereas rs2248374G homozygotes produce truncated transcript undergoing nonsense-mediated decay and therefore have no functional ERAP2 enzyme (28). Therefore, roughly every fourth individual in most human populations is devoid of ERAP2 activity. This, in many cases, is compensated by the presence of highly activity ERAP1 and *vice versa*. For example, in *ERAP2* rs2248374GG (no ERAP2) individuals, the ERAP1 allotype No. 2 homozygotes (high ERAP1 activity) are 14 times more frequent than in rs2248374AA homozygotes. On the other hand, the highest ERAP2 expression allotype is found more frequently in individuals that carry an intermediate or low-activity ERAP1 allotype (25). Generally, the higher the activity of ERAP1, the less frequently functional ERAP2 coexists with it in a haplotype and vice versa (27). Thus, a lack of functional ERAP2 apparently brings a need for higher activity of ERAP1. The close localization of both *ERAP* genes on chromosome 5q15 (29) favors complementing *ERAP1–ERAP2* haplotypes to provide at least one active ERAP. In addition, a rs75862629 SNP between both genes regulates mutually their expression in such a way that the rs75862629G allele is associated with high ERAP1 and low ERAP2 expression, and the opposite is observed with the A allele (30).

In most populations, the SNP rs2549782G>C,T (Lys392Asn) is in very strong LD with rs2248374, which decides whether functional ERAP2 is produced (28). The Asn392 variant has about 160 times higher activity than Lys392 (31). However, due to this strong LD, this variant is normally never produced except for very rare recombinants, and even in an exceptional population of Chileans, where such a recombinant is present in 2% of the population, Asn392/Asn392 homozygotes could not be detected (28). By using Asn392-overexpressing transfectants of the trophoblast cell line, Warthan etal. (32) found that these cells were killed by T and NK cells, and Lospinoso etal. (33) observed that such transfection caused downregulation of many genes important for cell survival, which together may explain the lack of Asn392 homozygotes in Chileans.

Other SNPs in *ERAP2* are also in very strong LD in humans (34). Therefore, two allotypes prevail in human populations: allotype A (with rs2248374A), a functional enzyme, and allotype B (with rs2234874G), not expressed in normal conditions (see *ERAP2 Isoforms*).

The lack of functional ERAP2 in roughly every forth human individual is not exceptional in nature. Most rodents do not even possess the *ERAP2* gene. Other mammals generally possess both *ERAP1* and *ERAP2* genes. Interestingly, multiple different combinations of *ERAP1* and *ERAP2* (and also *IRAP*) appear in other vertebrates; for example, about 60% of bony fish species possess *ERAP2*, but only 2% have the *ERAP1* gene (21).

## 4 Associations of rs2248374A>G With Human Disease

Similarly to ERAP1, ERAP2 polymorphisms also have an impact on the immunopeptidome, albeit with different results than the former, as stated in *Endoplasmic Reticulum Aminopeptidases*. Therefore, the lack of active ERAP2 enzyme in allotype B homozygotes results in changes in the immunopeptidome, as it was shown for HLA-B*27, associated with ankylosing spondylitis (35). However, the association of *ERAP2* with this disease appears independent of the presence of HLA-B*27, in contrast to the *ERAP1* association in epistasis with HLA-B*27 (36), which may speak to other functions of ERAP2 in addition to epitope production (see *ERAPS in the Modulation of Cytokine-Mediated Signaling, in the Regulation Of Blood Pressure, and in Sars-Cov-2 and HIV Infection*). Among other conditions where associations of *ERAP2* presence versus absence were observed, we may also list psoriasis, Crohn’s disease, hypertension, and birdshot chorioretinopathy (14, 17, 37). Similar associations were also described in gynecological diseases: preeclampsia (31, 38–40) and recurrent implantation failure after *in vitro* fertilization (41).

Interestingly, addition of recombinant full-length ERAP2 protein to cultures of peripheral blood mononuclear cells (from both allotype A and allotype B donors) infected with HIV-1 reduced viral replication by activation of cytotoxic CD8+ T cells (42). Moreover, even in CD8+ T-cell depleted peripheral blood mononuclear cell cultures, viral replication was reduced due to the activation of monocytes by ERAP2 (43).

## 5 ERAP2 Isoforms

Both ERAPs appear in several protein isoforms. Two ERAP1 isoforms resulting from alternative splicing, 19 exons (19E)- and 20 exons (20E)-containing transcript variants were described, whose expression is inversely dependent on the rs7063 SNP genotype (44). As ERAP1 is not the subject of this review, we will not go further here in this direction.

Although ERAP2 rs2248374GG individuals, as stated above, do not produce active aminopeptidase, it was recently found that during influenza infection, a truncated protein devoid of enzymatic activity was detected in these individuals. It has been named isoform 3 (Iso3) in opposition to Iso1, the normal functional ERAP2 present in rs2248374A-possessing individuals, and Iso2 and Iso4, truncated transcripts from rs2248374G undergoing nonsense-mediated decay and therefore giving no protein products (45) (**Table 1**). Later, Iso3 expression was confirmed also for other viral infections (HIV, human cytomegalovirus, SARS-CoV-2). Iso3 is apparently also present in bacterial infections, as it was also induced *in vitro* by lipopolysaccharide (46).

**Table 1:** ERAP2 isoforms (45, 46).

ERAP2 rs2248374 allele	Isoform	Function
A	Iso1	Normal transcript giving functional protein
G	Iso2	Truncated transcript undergoing nonsense-mediated decay
G	Iso3	Truncated transcript, induced by viral infection, giving truncated protein devoid of enzymatic activity
G	Iso4	Truncated transcript undergoing nonsense-mediated decay

It has been suggested that, as ERAP2/Iso3 possesses a domain required for homo- and heterodimer formation, it may possibly interfere with ERAP1 and ERAP2/Iso1 function in virus-infected cells (45, 46). It is not known yet whether such interactions positively or negatively affect antiviral immune responses.

## 6 ERAP2 Interactions With ERAP1

ERAP1 and ERAP2 may act separately, but about 30% of their molecules form heterodimers that may digest substrates faster than individual ERAPs (47, 48). Computational molecular dynamics calculations, based on experimentally determined homo-dimerization interfaces observed in crystal structures of ERAP2 or homologous enzymes, suggested the most likely ERAP1/ERAP2 heterodimerization topology. It involves the exon 10 loop (49), previously implicated in interactions between ERAP1 and the disulfide-bond shuffling chaperone ERp44. This latter interaction was postulated to be responsible for retaining ERAP1 (not possessing the ER retention signal) in the ER (50). The ERAP1/ERAP2 heterodimers produce fewer different peptide sequences than each of them separately, although in larger amounts; they leave only peptides resistant to degradation by each of them (51). Thus, on one hand, ERAPs produce, from longer precursors, peptides which may be bound and presented by HLA-I molecules; on the other hand, they may overtrim peptides, preventing them from being presented. Therefore, ERAP1 and ERAP2 may complement each other in epitope production or, on the contrary, mutually destroy peptides produced by each of them. Some peptides, sensitive to ERAP1-mediated overtrimming, may be protected from it if bound by ERAP2, particularly in the context of an ERAP1 allotype with low activity (27).

## 7 Associations of *ERAP1–ERAP2* Haplotypes With Human Disease

As stated above, a simultaneous activity of ERAP1 and ERAP2 (and their heterodimer) may result in a different immunopeptide repertoire than that of each enzyme alone. It is not surprising, then, that *ERAP1–ERAP2* haplotypes were described as being associated with several human diseases stronger than each gene separately.

Ankylosing spondylitis is strongly associated with HLA-B*27—90% of patients are positive (52–54), versus less than 10% in the healthy Europeans, e.g., in Poles (55). Nevertheless, only a small fraction of HLA-B*27-positive individuals get the disease. Several groups of researchers examined the role of ERAP1 and ERAP2 as ankylosing spondylitis risk factors in addition to HLA-B*27 (reviewed by 17, 18, 26, 56–58). The role of ERAPs in shaping the HLA-B*27:05 peptide repertoire was examined by the Lopez de Castro group: both highly active ERAP1 allotype and the presence of ERAP2 affected the HLA-B*27 immunopeptidome, but their effects were distinct, suggesting that they act here as separate, independent entities (17, 19, 35). The same haplotype (highly active ERAP1 and the presence of ERAP2) was described as a risk factor, while ERAP1 with low activity and a lack of functional ERAP2 were protective (19, 24, 35). Interestingly, while ERAP1 was associated with ankylosing spondylitis only in HLA-B*27-positive individuals, ERAP2 was associated with this disease independently from HLA-B*27 (17, 18, 26, 56–58), which may suggest it participates in another activity in addition to peptide trimming (see the next section). Moreover, we observed that the same SNP combination in *ERAP1* was associated with risk or protection from AS or not, depending on the absence or presence of ERAP2 (59).

Psoriasis, a relatively common skin disease, is associated with HLA-C*06:02, although some 30% of patients do not possess this allele (19, 26). A genome-wide association study of psoriasis identified eight previously unreported regions of the genome, among them *ERAP1* (60). Although originally the *ERAP1* association (ERAP2 was not examined there) with psoriasis was claimed to be independent of HLA-C*06:02 (61), we recently observed different associations of *ERAP1–ERAP2* haplotypes, depending on the presence versus absence of the HLA-C*06:02 gene: namely, low activity ERAP1 together with ERAP2 was protective in HLA-C*06:02-positive individuals but associated with disease risk in HLA-C*06:02-negatives. The same ERAP1 without ERAP2 had no effect in HLA-C*06:02-positive patients but was protective in HLA-C*06:02-negative patients. Another ERAP1 allotype, with high activity, was protective in both HLA-C*06:02-positive and HLA-C*06:02-negative patients if accompanied by the presence of ERAP2, but brought risk in HLA-C*06:02-positive patients and had no effect in HLA-C*06:02-negative patients when ERAP2 was absent (62). This suggests different trimming requirements of HLA-I molecules associated with psoriasis in HLA-C*06:02-positive and HLA-C*06:02-negative patients, respectively. Three different autoantigens have already been implicated in psoriasis: cathelicidin LL37, an antimicrobial peptide (63); ADAMTSL5, a melanocyte-derived autoantigen (64); and keratin 17, homologous to streptococcal M-proteins (65). All three are overexpressed in psoriatic skin. LL37 was recognized by CD8+ T cells in the context of HLA-C*06:02 or HLA-A*11, depending on the HLA background of the patient (63). ADAMTSL5 was HLA-C*06:02-presented (64). Keratin 17 induced stronger CD8+ T-cell responses in HLA-C*06:02-positive patients than in HLA-C*06:02-negative ones (65). Therefore, psoriatogenic autoantigens are generally presented more effectively by HLA-C*06:02 than by other HLA-I allotypes, but the latter may also play a role in this disease, as is obviously the case in HLA-C*06:02-negative individuals. A contribution of other psoriatogenic antigens in HLA-C*06:02-negative cases cannot be excluded.

Another skin disease, atopic dermatitis, as an allergic disease, is mainly associated with HLA class II alleles (66). However, HLA-I alleles may play a role in disease outcome (67), and indeed, we found KIR2DS1 (a receptor of NK cells recognizing HLA-C) was associated with protection against atopic dermatitis (68). We also found an association of rs26618 in *ERAP1*, a SNP affecting enzyme activity, with this disease (69). Although *ERAP2* presence/absence had no effect on disease susceptibility, homozygosity for the ERAP2 presence (i.e., rs2248374A/A) together with rs26618C/C resulted in a higher risk of atopic dermatitis than rs26618 alone (70).

A rare disease, birdshot chorioretinopathy, manifesting as small light-colored fundus spots on the retina, scattered in a pattern like birdshot from a shotgun, is a condition most strongly associated with HLA-I, namely with HLA-A*29 (virtually all patients are HLA-A*29-positive). It affects mostly Europeans or patients of European descent. Although HLA-A*29 is not infrequent in some non-European populations, birdshot chorioretinopathy is much less frequent there. The *ERAP1* rs2287987 or *ERAP2* rs10044354 SNPs are present in 90% of patients, and the rs2287987-rs10044354 haplotype is in 50% (and twice less frequent in controls). This haplotype is less common in non-Europeans, which may explain why the disease is afflicting mostly people of European descent (27). Both ERAP1 and ERAP2 polymorphisms have an influence on the HLA-A*29-bound immunopeptidome (19), providing an explanation for their association with birdshot chorioretinopathy.

Also, in nonsmall cell lung cancer some ERAP1 allotypes were associated with cancer risk, protection, or without effect, depending on the presence versus absence of ERAP2 (ERAP2 separately was without effect). Interestingly, these effects were frequently different in smokers and never smokers (71).

Thus, in many clinical conditions, the effect of the combined activities of ERAP1 and ERAP2 on susceptibility to disease and its outcome is different from the effects of each enzyme alone. Therefore, typing for polymorphisms of both *ERAP1* and *ERAP2* may have a predictive value for therapy utilizing ERAP inhibitors.

## 8 ERAPs in the Modulation of Cytokine-Mediated Signaling, in the Regulation of Blood Pressure, and in SARS-CoV-2 and HIV Infection

In addition to their role in trimming HLA-I-presented peptides (including SARS-CoV-2 peptides) in the endoplasmic reticulum, ERAPs also perform other functions after secretion from cells. There, they may trim cell surface receptors, e.g., cytokine receptors such as tumor necrosis factor receptor 1, interleukin 6 receptor alpha, and interleukin 1 receptor II (72, 73) and plasma proteins (74 and references therein). Although soluble receptors do not transmit signals directly, they nevertheless can affect ligand binding and activation of membrane receptors and therefore indirectly modulate cellular signaling (75). Solubilized receptors can affect cytokine signaling far away from their site of origin (56 and references therein). In this way, ERAPs contribute to the regulation of several physiological processes in addition to antigen presentation. Among other substrates, ERAPs cleave angiotensins engaged in keeping the balance between hyper- and hypotension (see below).

Blood pressure is regulated by the renin-angiotensin system using a complicated array of proteins, enzymes cleaving them, and receptors. Briefly, angiotensinogen is cleaved by renin to angiotensin I, which is further converted by angiotensin-converting enzyme (ACE) to angiotensin II. This protein binds the ACE/Ang II/Ang II receptor type 1 (AT1R), resulting in vasoconstriction, hypertension, and inflammation. ACE2 counter-regulates this process *via* the cleaving of angiotensin I to angiotensin 1–7 and angiotensin II to angiotensin 1–9, which binds the Mas receptor, initiating vasodilation, hypotension, and anti-inflammatory activity (76, 77). ERAPs also contribute to the regulation of blood pressure. Both ERAPs, in addition to their intracellular role in antigen presentation, after secretion regulate hypertension by trimming angiotensin II to angiotensin III (ERAP1) and angiotensin III to angiotensin IV (ERAP1 and ERAP2) which then bind to receptors AT2R and AT4R, respectively, stimulating vasodilation and counteracting hypertension (29, 50, 78–80). These angiotensins consist of eight (angiotensin II: DRVYIHPF), seven (angiotensin III: RVYIHPF), and six (angiotensin IV: VYIHPF) amino acids (77). These lengths seem to be too short for ERAP1 but may still be acceptable for ERAP2. In addition, the sequence of angiotensin II starts with an Asp-Arg dipeptide and that of angiotensin III with Arg, which are not efficiently excised by ERAP1 (18, 22). Therefore, their trimming by ERAP1, although experimentally proven, is hard to explain. Perhaps it is indeed performed, but slowly and with low efficiency. Angiotensin III sequence starts with Arg, which is a favorable substrate for ERAP2 (18, 22), so its conversion to angiotensin IV by this aminopeptidase is understandable.

ERAP2/Iso3, and also ERAP1 low activity allotypes, cannot perform these functions, thus moving equilibrium to stimulation of AT1R which causes vasoconstriction and hypertension (81). It is conceivable that the primary function of ERAPs (and IRAP) was regulation of blood pressure through the renin–angiotensin system and that epitope production by peptide trimming evolved later, paralleling the evolution of the specific immune system, including the major histocompatibility complex (21). Interestingly, however, in a recent review of genetic polymorphisms affecting the regulation of blood pressure, Cuevas etal. (76) do not mention ERAPs at all.

During SARS-CoV-2 infection, the virus uses cell membrane angiotensin I-converting enzyme 2 (ACE2) as a receptor to bind to the cell; the complex of the virus with ACE2 is internalized, viral RNA is released to the cytosol, and the viral particles are produced and released to infect other cells. The decrease of ACE2 from the cell surface by endocytosis and enzymatic degradation results in ACE/ACE2 imbalance and dominance of the ACE-mediated pathway leading to hypertension, acute lung injury, and multiorgan damage. Defective variants of ERAPs (i.e., low activity ERAP1 allotypes and lack of functional ERAP2) exacerbate the impact of SARS-CoV-2 on hypertension and organ damage. On the other hand, active ERAP variants may counter-balance SARS-CoV-2-mediated pathogenesis (81).

It should be noted here that secreted ERAP2 was postulated also to play a role in HIV infection: by trimming N-terminal arginine from extracellular substrates, it increases free arginine concentration, essential for synthesis of nitric oxide, an important biological messenger. Thus, ERAP2 may affect protection against HIV in this way in addition to its role in immunopeptidome production, contribution to the regulation of hypertension, and a possible effect of interactions of truncated ERAP2/Iso3 with ERAP1 or with untruncated ERAP2/Iso1 (42, 43, 46).

## 9 Conclusions

The original finding that functional ERAP2 protein is absent in 25% of individuals who mostly remain healthy, and that the immune system of the mouse copes well without ERAP2, suggested that it is basically unimportant and plays only a subsidiary role for ERAP1. However, ERAP2 activity may support effective immune response to a pathogen. On the other hand, the lack of ERAP2 may, in many instances, prevent it from overtrimming antigenic peptides produced by ERAP1, which are necessary to fight infection or contribute to autoimmune disease. In addition, the defective ERAP2 allele is not without function in immune defense as, in viral infections, it produces truncated protein isoform possibly interfering with ERAP1 and functional (allotype A) ERAP2. In addition, ERAPs secreted from the cells may trim cell surface cytokine and growth factor receptors, affecting immune response indirectly, and this activity may also be dependent on their polymorphisms. They may also regulate blood pressure and influence the severity of the coronavirus disease 2019 (COVID19). Therefore, some ERAP1–ERAP2 combinations may be protective against one disease but carry a risk for another. So, for ERAP2, the question “to be or not to be,” i.e., whether it is better to have ERAP2 or not, may depend on the genetic background of an individual (including, first and foremost, his/her *HLA* genotype) as well as on environmental factors. In some circumstances, the presence of an active ERAP2 variant may be beneficial, but in others, it may be harmful. Some of us, due to bad luck, improper genetic constitution (among it, the presence or absence of ERAP2) and environmental influence, contract serious or even fatal diseases, but our species as a whole survives even the worst pandemics such as cholera, plague, AIDS, or, now, COVID-19. For those less lucky, whose genetic (including *ERAP2*) and environmental factors predispose them to a disease, ERAP2 inhibitors may in some circumstances, become helpful if the patients possess this enzyme in its active form and if it contributes to an unfavorable condition.

## Author Contributions

The author confirms being the sole contributor of this work and has approved it for publication.

## Funding

The author’s work on associations of ERAPs with human diseases was supported by grants 14/2015, 14/2016, 14/2018, 14/2019, 14/2020, 14/2021, and 14/2022 from the Hirszfeld Institute of Immunology and Experimental Therapy, the grants N402685040, OPUS 8 2014/15/B/NZ5/03517, and 2018/29/N/NZ5/00940 from the Polish National Science Centre, and the Medical University of Łódź research study 503/5 064 01/503 1.

## Conflict of Interest

The author declares that the research was conducted in the absence of any commercial or financial relationships that could be construed as a potential conflict of interest.

## Publisher’s Note

All claims expressed in this article are solely those of the authors and do not necessarily represent those of their affiliated organizations, or those of the publisher, the editors and the reviewers. Any product that may be evaluated in this article, or claim that may be made by its manufacturer, is not guaranteed or endorsed by the publisher.

## Footnotes

1 To avoid confusion of the reader with multiple names of these enzymes encountered in the literature, we should note that ERAP1 is also designated adipocyte-derived leucine aminopeptidase (A-LAP), puromycin-insensitive leucine-specific aminopeptidase (PILSAP), ER associated with antigen presentation—this is used for mouse enzyme (ERAAP), and aminopeptidase regulator of TNFR1 shedding (ARTS-1). ERAP2 is also referred to as leucocyte-derived arginine aminopeptidase (L-RAP). Insulin-regulated aminopeptidase (IRAP) is also named placental leucine aminopeptidase (PLAP) and leucyl-cystinyl aminopeptidase (LNPEP).
